# Differential effects of leptin on energy metabolism in murine cell models of metastatic triple negative breast cancer

**DOI:** 10.1186/s13098-024-01535-1

**Published:** 2024-11-28

**Authors:** Chaehyun Yum, Chaylen Andolino, Marjorie Anne Layosa, Michael Coleman, Stephen D. Hursting, Dorothy Teegarden

**Affiliations:** 1 Department of Nutrition Science, Interdepartmental Nutrition Program, Purdue University, West Lafayette, IN 47907 USA; 2 Purdue University Institute for Cancer Research, Purdue University, West Lafayette, IN 47907 USA; 3 Department of Nutrition, University of North Carolina, Chapel Hill, NC 27516 USA; 4 Lineberger Comprehensive Cancer Center, University of North Carolina, Chapel Hill, NC 27516 USA

**Keywords:** Breast cancer, Leptin, Glucose, Fatty acid

## Abstract

**Background:**

Leptin, an energy balance regulator secreted by adipocytes, increases metastatic potential of breast cancer cells. The impact on cancer cell metabolism remains unclear given that most studies of leptin and breast cancer cell metabolism utilize supraphysiological glucose concentrations.

**Methods:**

Using two murine models of metastatic triple-negative breast cancer (TNBC) differing in genetic alterations (4T1: p53 and Pik3ca mutations; metM-Wnt^lung^: increased Wnt signaling) and cultured in physiological (5 mM) glucose media, we tested the hypothesis that leptin increases migration of metastatic breast cancer cells through regulation of glucose metabolism.

**Results:**

Our results showed that leptin treatment, compared with vehicle, increased cell migration in each cell line, with decreased leptin receptor (*Ob-R*) mRNA expression in 4T1, but not metM-Wnt^lung^, cells. AMP-activated protein kinase (AMPK) was activated in 4T1 with leptin treatment but decreased in metM-Wnt^lung^. Leptin decreased fatty acid synthase (*Fasn*) and carnitine palmitoyltransferase 1a (*Cpt1a*) mRNA expression in 4T1 cells but increased their expression in metM-Wnt^lung^ cells. Fatty acid oxidation was not necessary for leptin-induced migration in either cell line. Leptin increased palmitate synthesis from glucose in metM-Wnt^lung^, but not 4T1 cells. Moreover, although leptin increased glucose transporter 1 (*Glut1*) mRNA expression in both cell lines and inhibition of glycolysis blocked leptin-induced migration in metM-Wnt^lung^, but not 4T1 cells.

**Conclusion:**

Taken together, these results demonstrate that at physiological glucose concentrations, leptin increases migration of 4T1 and metM-Wnt^lung^ cells via shared and distinct effects on energy metabolism, suggesting that the type of TNBC genetic alteration plays a role in differential metabolic regulation of leptin-induced migration.

## Background

Leptin, encoded by the obese (*ob*) gene, is an adipokine that regulates whole-body energy balance and appetite. Leptin is also involved in regulating immune responses, reproduction, and proliferation of many cell types, including breast cancer cells [1]. Molecular action of leptin is mediated through the transmembrane leptin receptor (Ob-R). Canonically, leptin binds to the leptin receptor and activates janus kinase (JAK)/signal transducer and activator of transcription (STAT) [2] and mitogen-activated protein kinase (MAPK) signaling pathways [3, 4], whose downstream effects assist in promoting cancer metastasis through increasing migration and invasion. Both leptin and the leptin receptor are overexpressed in primary breast cancer tumors and lymph node metastases, suggesting that this signaling may contribute to cancer progression [5]. Nonetheless, the roles of cellular energy metabolism in leptin-induced pro-cancer processes are not fully established.

Reprogramming of lipid metabolism is a recognized hallmark of breast cancer aggressiveness [6]. Fatty acids (FA), either endogenously synthesized, exogenously taken up, or released from lipid droplet storage, can serve numerous functions in the cell, including as a source of energy via fatty acid oxidation (FAO) or as signaling molecules [7]. These functions of FAs in part support cancer progression and migration. Overexpression of fatty acid synthase (FASN) occurs in multiple cancers [8], which indicates a potential role of dysregulated fatty acid synthesis in cancer metabolism. In addition, leptin decreases FASN and increases FAO in MCF-7 breast cancer cells [9], suggesting that leptin-mediated changes in lipid metabolism may support cancer progression.

Glucose and its metabolites serve as critical energy substrates as well as precursors for lipids, thereby linking glucose and lipid metabolism. Additionally, glucose enhances leptin signaling in human fibrosarcoma cells [10], suggesting a potential role of glucose in affecting the actions of leptin. AMP-activated protein kinase (AMPK) is a nutrient energy sensor that can regulate glucose and lipid metabolism in response to energy status [11]. When there is a deficit of energy, the AMP/ATP ratio increases and AMPK is activated by AMPK kinases, leading to the activation of catabolic processes. Conversely, AMPK is inactivated when energy is sufficient. Decreased AMPK activation is associated with higher grades of breast cancer [12], suggesting that AMPK is critical in the ability of cells to metabolically adjust to the adverse conditions experienced during metastasis. Most in vitro research to date examining the effects of leptin on breast cancer cell migration employed supraphysiological glucose concentrations [13, 14], and has reported that AMPK is activated by leptin treatment in breast cancer cells [13, 15].

Using two murine models of metastatic triple-negative breast cancer (TNBC) differing in their genetic alterations, we investigated the impact of leptin signaling on breast cancer metastasis under physiological (5 mM) glucose conditions. The murine 4T1 cells have p53 and Pik3ca mutations, form primary tumors when implanted into the mammary gland of BALB/c mice, metastasize to the lung, liver, brain, and bone [16], and mimic stage IV breast cancer [17]. Developed from the nonmetastatic M-Wnt cell line through serial passage in mice, the metM-Wnt^lung^ cells have increased Wnt signaling, form tumors and metastasize to the lung in vivo when transplanted into syngeneic mice [18]. We hypothesized that leptin alters energy metabolism in physiological conditions, and that this change is necessary for leptin-induced migration. By clarifying leptin’s regulation of lipids and glucose metabolism in breast cancer cell migration, this work may inform development of therapies to reduce obesity-associated metastatic breast cancer.

## Materials and methods

### Chemical and reagents

Dulbecco’s Modification of Eagle’s Medium (DMEM) was obtained from Corning. Fetal bovine serum (FBS), trypsin, and penicillin/streptomycin were obtained from Life Technologies, Gibco-BRL. Recombinant leptin and 5-aminoimidazole-4-carboxamide ribonucleotide (AICAR) were purchased from Peprotech. 2-deoxyglucose (2DG), Etomoxir, and TVB-3166 were obtained from Sigma-Aldrich.

### Cell culture

The metM-Wnt^lung^ cell line was generated by serial transplantation of nonmetastatic M-Wnt cell line as described previously [18]. Cells (4T1 and metM-Wnt^lung^) were cultured in 5 mM glucose and 4 mM glutamine containing DMEM without sodium pyruvate, with 10% FBS, 100 units/mL penicillin, and 100 µg/ml streptomycin in a humidified environment at 37 °C with 5% CO_2_. Cells were treated for 4 days with either vehicle (bovine serum albumin (BSA) final concentration 0.0001%) or leptin (300 ng/ml). Leptin (1000X) was dissolved in 0.1% BSA as per manufacturer’s instruction.

### Migration assay

The cells were pretreated with 300 ng/mL of leptin or vehicle for 4 days before replating in serum-free medium into 8 μm pore transwell inserts (Corning). Transwell inserts were placed into 10% FBS-containing media with the indicated glucose concentration, with no additional treatment present. After 15–24 h of incubation for metM-Wnt^lung^ and 4T1 cells, respectively, the bottom of the transwells were fixed with 100% methanol and stained with 2% crystal violet in ethanol. The cell number was counted in 5 different fields per transwell and migration was quantified using the average sum of cells/field.

### RNA isolation and analysis

RNA was isolated with TriReagent (Molecular Research Center) and reverse transcription to cDNA conducted using MMLV reverse transcriptase (Promega). mRNA level was determined using qPCR and data are normalized to 18 S level. Real-time quantitative PCR was performed using LightCyler 480 SYBR Green I Master Mix (Roche). The comparative Ct method (2^−ΔCt^) was used to calculate relative gene expression. Data are expressed as fold change relative to vehicle. Primers used are shown in Table 1.

**Table 1:** Primers used in the qPCR analysis of mRNA level

Genes	Forward Primer 5’-3’	Reverse Primer 5’-3’
*Acly*	ACCCTTTCACTGGGGATCACA	GACAGGGATCAGGATTTCCTTG
*Cpt1a*	CTGCAGTCGGTCACCACT	ACACCCACCACCACGATAAG
*Cpt1b*	GCACACCAGGCAGTAGCTTT	CAGGAGTTGATTCCAGACAGGTA
*Fasn*	ACCACTGCATTGACGGCCCGG	GGGTCAGGCGGGAGACCGAT
*Glut1*	GGCTTGCTTGTAGAGTGACGA	GTGAGTGTGGTGGATGGGAT
*HK2*	TGATCGCCTGCTTATTCACGG	AACCGCCTAGAAATCTCCAGA
*Ob-R*	CCTCTTGTGTCCTACTGCTCG	GAAATTCAGTCCTTGTGCCCAG
*18 S*	ATCCCTGAGAAGTTCCAGCA	CCTCTTGGTGAGGTCGATGT

### Triacylglycerol (TAG) level

Lipids were extracted from leptin or vehicle-treated cells using 3:2 hexane: isopropanol after washing with Tris-buffered saline solution containing 0.2% fatty acid free BSA. Extracted samples were dried, resuspended in 1% TritonX-100 in chloroform, dried again, and solubilized in 2% Triton X-100 in water. The triacylglycerol (TAG) level was assessed spectrophotometrically, according to the manufacturer’s instructions (Wako Diagnostics). Results are normalized to protein assessed by the bicinchoninic acid (BCA) protein assay (Sigma-Aldrich).

### Fatty acid (FA) synthesis

During the last 24 h of treatment, 4T1 and metM-Wnt^lung^ cells were incubated with 10 mM [^13^C_2_] acetate (Sigma-Aldrich) or glucose-free DMEM containing a 1:1 mixture of D-[U-^13^C]glucose (Sigma-Aldrich) and non-labeled D-glucose at a final glucose concentration of 5 mM (0.9 g/L). Cells were harvested into lysis buffer, and lipid hydrolysis, extraction, derivatization, and analysis by liquid chromatography-tandem mass spectrometry (LC-MS/MS) were performed as previously described [19]. Substrate incorporation into palmitate (16:0) and stearate (18:0) FAs was assessed as area under the curve (AUC). Weighted analysis was conducted to calculate the number of ^13^C-labeled carbons for each peak. The data are calculated by dividing the sum of weighted AUC to the sum of all AUC times 100, as percent total palmitate or stearate from ^13^C-glucose or ^13^C-acetate.

### FA uptake

To measure FA uptake, cells were washed with calcium/magnesium-free phosphate-buffered saline, incubated with 10 µmol/L BODIPY FL C16 (4,4-difluoro-5,7-dimethyl-4-bora-3a,4a-diaza-s-indacene- 3-hexadecanoic acid; Life Technologies) in 0.1% FA-free BSA in Hanks balanced salt solution (Gibco–Life Technologies) for 5 min, and washed twice with an ice-cold solution of 0.2% FA-free BSA in Hanks balanced salt solution. Fluorescence was measured using a Synergy H1 Multi-Mode Reader (BioTek Instruments, Inc). The relative BODIPY FL C16 uptake is expressed as fluorescence intensity per well normalized to protein.

### Western blotting

After treatment, cells were washed with PBS and harvested on ice into radioimmunoprecipitation assay (RIPA) buffer containing 1% each of protease inhibitor and phosphatase inhibitor cocktails (Sigma-Aldrich). Protein concentration of the cell lysate was measured by BCA protein assay, separated by sodium dodecyl sulfate polyacrylamide gel electrophoresis on 10% polyacrylamide gels (Bio-Rad Laboratories) and transferred onto nitrocellulose membranes (Bio-Rad Laboratories). After blocking, membranes were incubated with rabbit anti-AMPKα, rabbit anti-phospho-AMPKα (Thr172), or rabbit anti-β-actin (Cell Signaling) overnight. The membrane was incubated with IRDye 680RD Donkey secondary antibody System (Li-Cor Biosciences), and imaged and quantified with Li-Cor Odyssey Imaging System (Li-Cor Biosciences).

### Intracellular pooled sizes measurement by GC-MS

Cells (4T1 and metM-Wnt^lung^) were treated with leptin or vehicle for 4 days. After the incubation period, cells were harvested into 70% ethanol at 70 °C. The internal standard norvaline (1 ug) was added to each sample and the samples were incubated for 5 min at 95 °C. After centrifugation, the supernatant was evaporated under nitrogen gas. The samples were derivatized using methoxylamine hydrochloride in pyridine, following a previously described method [20] and protein level assessed in the cell pellet (BCA). Derivatized metabolites were analyzed using gas chromatography-mass spectrometry (GC-MS). A TG-5MS gas chromatography column and Thermo TSQ 8000 triple quadrupole mass spectrometer were utilized. Mass spectra data were acquired using Chromeleon 7 software from ThermoFisher. Intracellular pool sizes were calculated by dividing the total metabolite AUC by norvaline and protein amount in the cell pellet to normalize for recovery variation and cell quantity.

### Statistical analysis

Values are presented as mean ± SEM. Results are expressed compared to the vehicle and analyzed by two-tailed Student’s t-tests. Where letters are provided, analysis was conducted using analysis of variance (ANOVA). Comparisons where *P* < 0.05 were considered statistically significant.

## Results

**Fig. 1:**
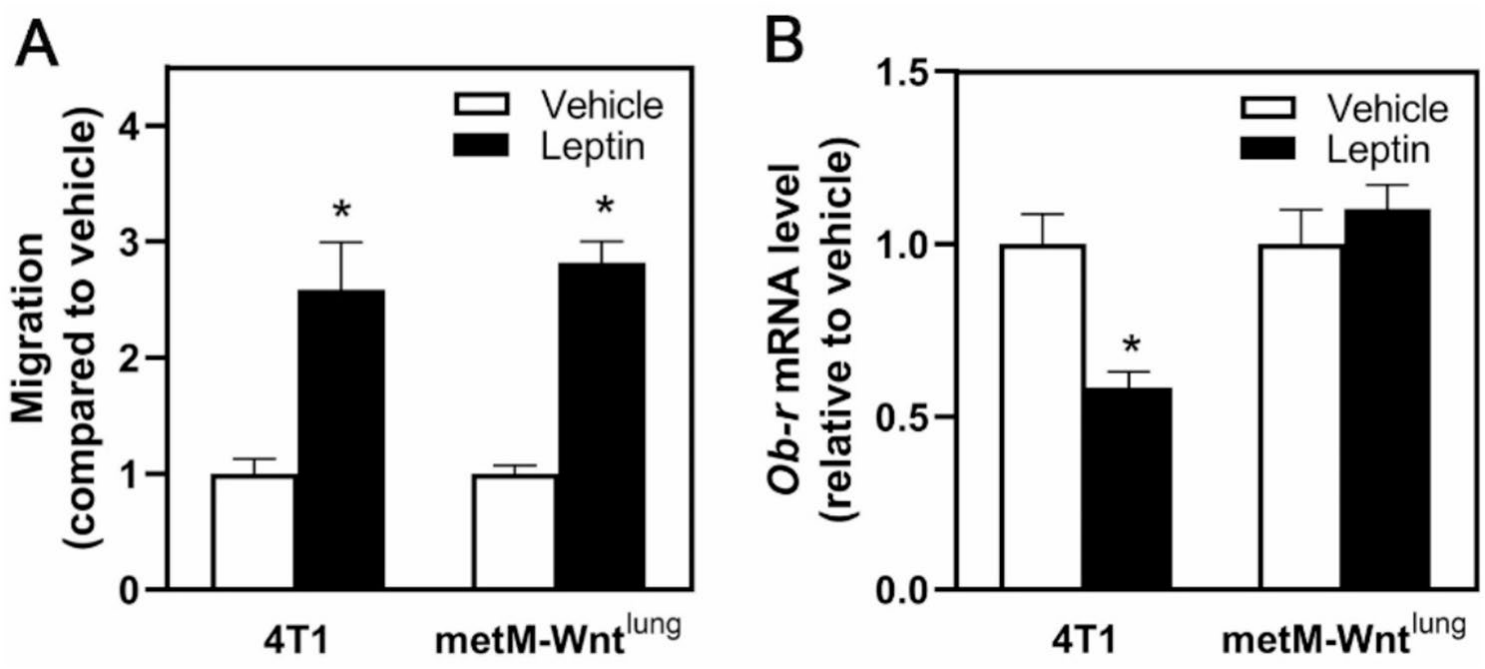
Leptin Effect on Migration of Metastatic Breast Cancer Cells. (**A**) Both 4T1 and metM-Wnt^lung^ cells were pretreated with leptin (300 ng/ml) for 4 days and replated in serum free medium into 8 μm pore transwell inserts and migration assessed. (**B**) Following leptin treatment or vehicle, mRNA level was measured in 4T1 and metM-Wnt^lung^ cells by qPCR. Values are mean ± SEM. Asterisk indicates a significant difference relative to vehicle (*p* < 0.05) of the same cell type

The impact of leptin on migration in 5 mM glucose medium was assessed in murine triple negative metastatic 4T1 and metM-Wnt^lung^ breast cancer cell lines. Leptin pretreatment, compared to vehicle, increased migration in both 4T1 and metM-Wnt^lung^ cells (Fig. 1A). Leptin also decreased leptin receptor (*Ob-R*) mRNA expression in 4T1, but not in metM-Wnt^lung^, cells (Fig. 1B), suggesting differential responses to leptin treatment in these cell lines.

**Fig. 2:**
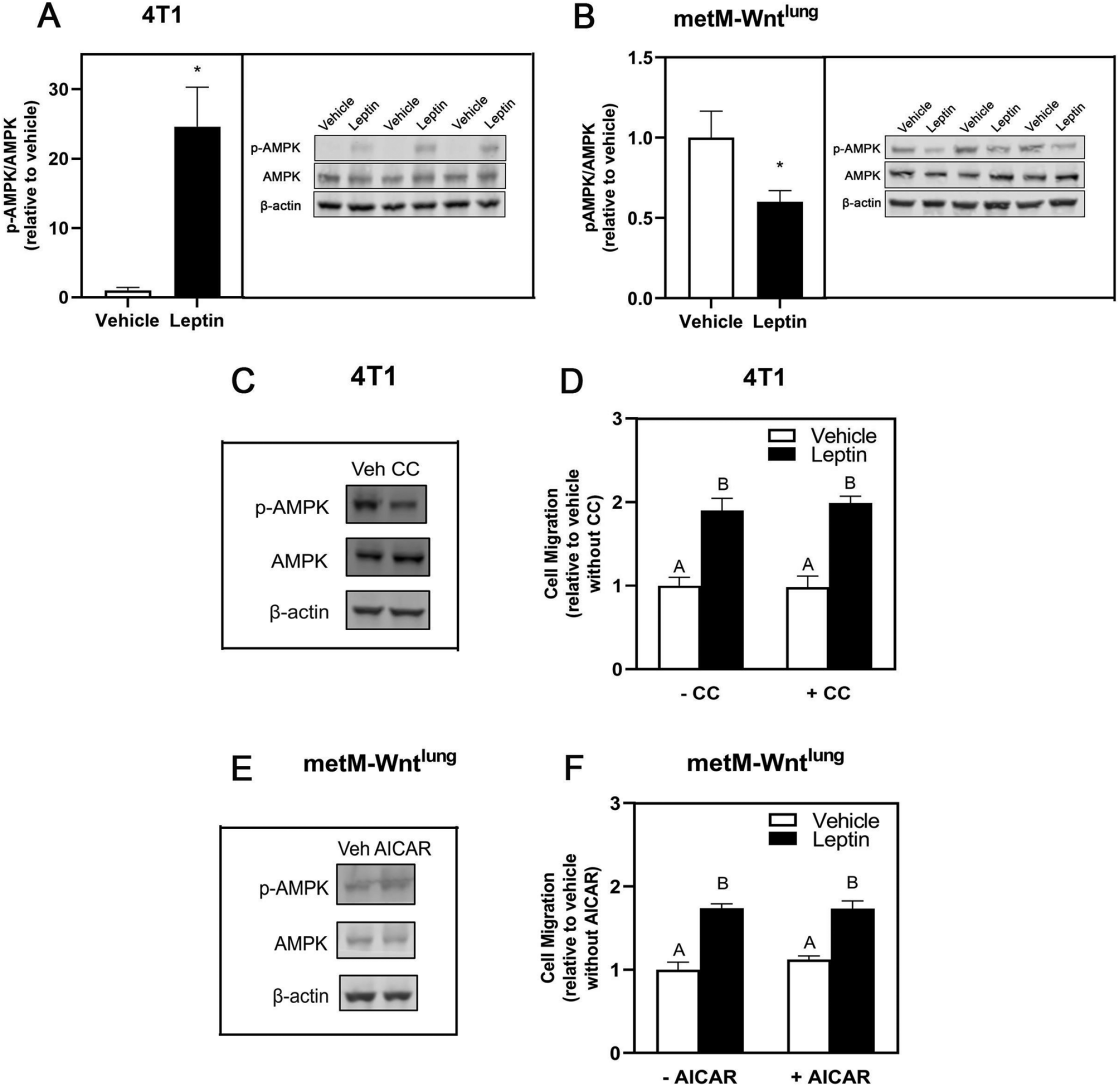
Leptin Regulation of AMPK Activation in Metastatic Breast Cancer Cells. Representative blots and quantification of p-AMPK and AMPK protein level with vehicle (Veh) or leptin treatment by Western blot with β-actin as a loading control in 4T1 (**A**) and in metM-Wnt^lung^ cells (**B**). Representative blots of p-AMPK and AMPK protein level with or without the AMPK inhibitor compound C (CC) (1 µM for 2 days) in 4T1 cells (**C**) and with or without the AMPK activator (AICAR) (1 mM for 2 days) in metM-Wnt^lung^ cells (**E**). (**D**) The 4T1 cells were pretreated with leptin for 4 days before replating in serum free medium with or without the AMPK inhibitor compound C (CC) (1 µM) into transwell inserts and migration assessed. (**F**) The metM-Wnt^lung^ cells were pretreated with leptin or vehicle for 4 days. During the last 24 h of treatment, cells were incubated with AICAR (1 mM) followed by replating in serum free medium with or without AICAR (1 mM) into transwell inserts and migration assessed. Values are mean ± SEM. Asterisk indicates a significant difference relative to vehicle (*p* < 0.05) of the same cell type. Groups with different letters are significantly different (*p* < 0.05) assessed by ANOVA

Given the high metabolic demands of cellular migration, we explored whether AMPK mediates the observed leptin-induced migration. Interestingly, p-AMPK/AMPK was increased in 4T1 cells with leptin treatment (Fig. 2A) but was decreased in metM-Wnt^lung^ cells (Fig. 2B), suggesting differential metabolic responses in these two cell lines following leptin treatment. To determine the sufficiency of AMPK signaling to inhibit leptin-induced migration, we treated 4T1 cells with an AMPK inhibitor (compound C) and metM-Wnt^lung^ cells with an AMPK activator (AICAR) with or without leptin treatment. While compound C inhibited p-AMPK/AMPK in 4T1 cells (Fig. 2C), no effect of the compound C on leptin-induced migration in 4T1 cells was observed (Fig. 2D), despite increased AMPK activity. Additionally, AMPK activator AICAR reduced AMPK activity (Fig. 2E) but did not affect leptin-induced migration in metM-Wnt^lung^ cells (Fig. 2F). Together, these results suggest that although AMPK signaling is differentially regulated, it is not required for leptin-induced migration in either 4T1 or metM-Wnt^lung^ cells.

**Fig. 3:**
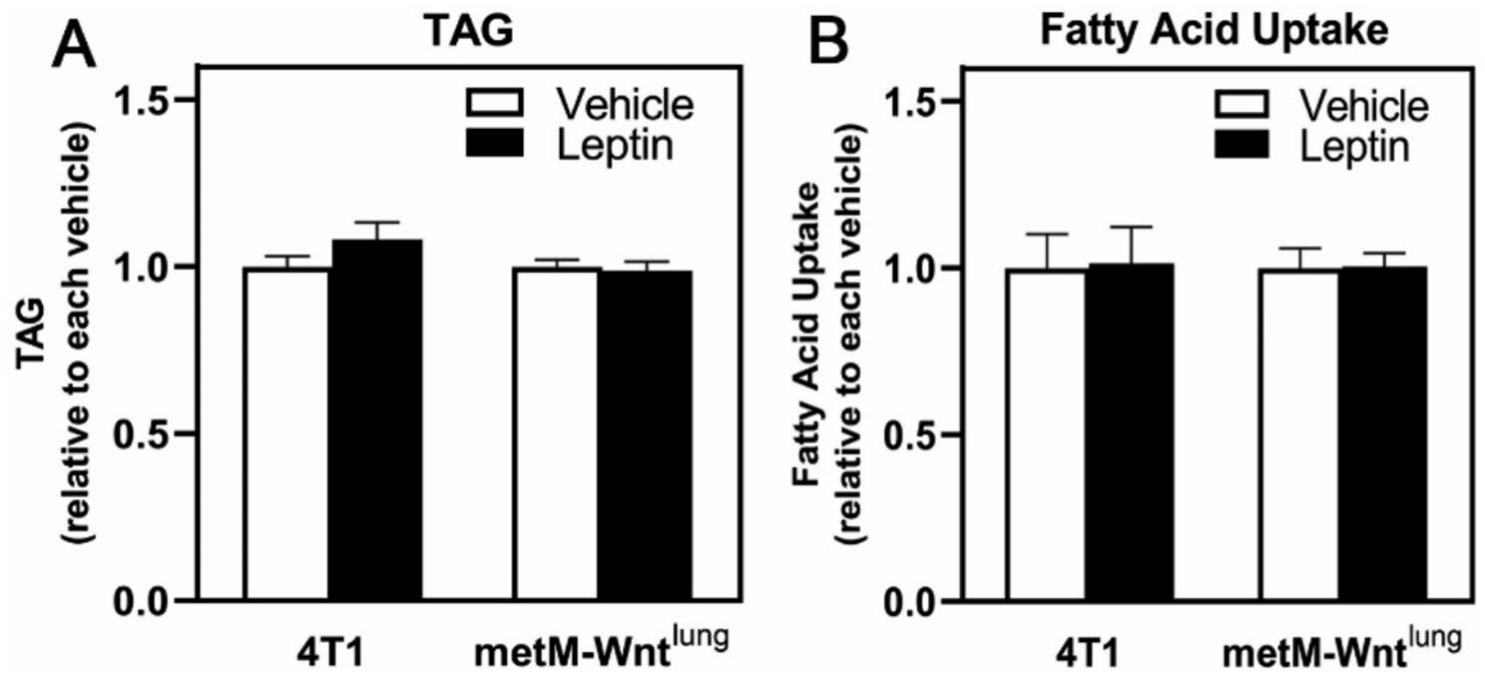
Leptin Regulation of TAG Level and FA Uptake in Metastatic Breast Cancer Cells. 4T1 and metM-Wnt^lung^ cells were treated with vehicle or leptin for 4 days. (**A**) TAG level was assessed and normalized to total protein per well. (**B**) Fatty acid uptake was determined following incubation with BODIPY FL-palmitate. The relative BODIPY FL-palmitate uptake is expressed as fluorescence intensity normalized to protein. No significant differences were noted, and values are mean ± SEM

Leptin is a potent regulator of fatty acid metabolism, including the neutral lipid TAG [21], therefore we assessed TAG levels and FA uptake. Leptin treatment did not change TAG levels or palmitic acid uptake in either cell line (Fig. 3A-B), suggesting that leptin does not alter the contribution of exogenous lipids to cellular lipid pools.

**Fig. 4:**
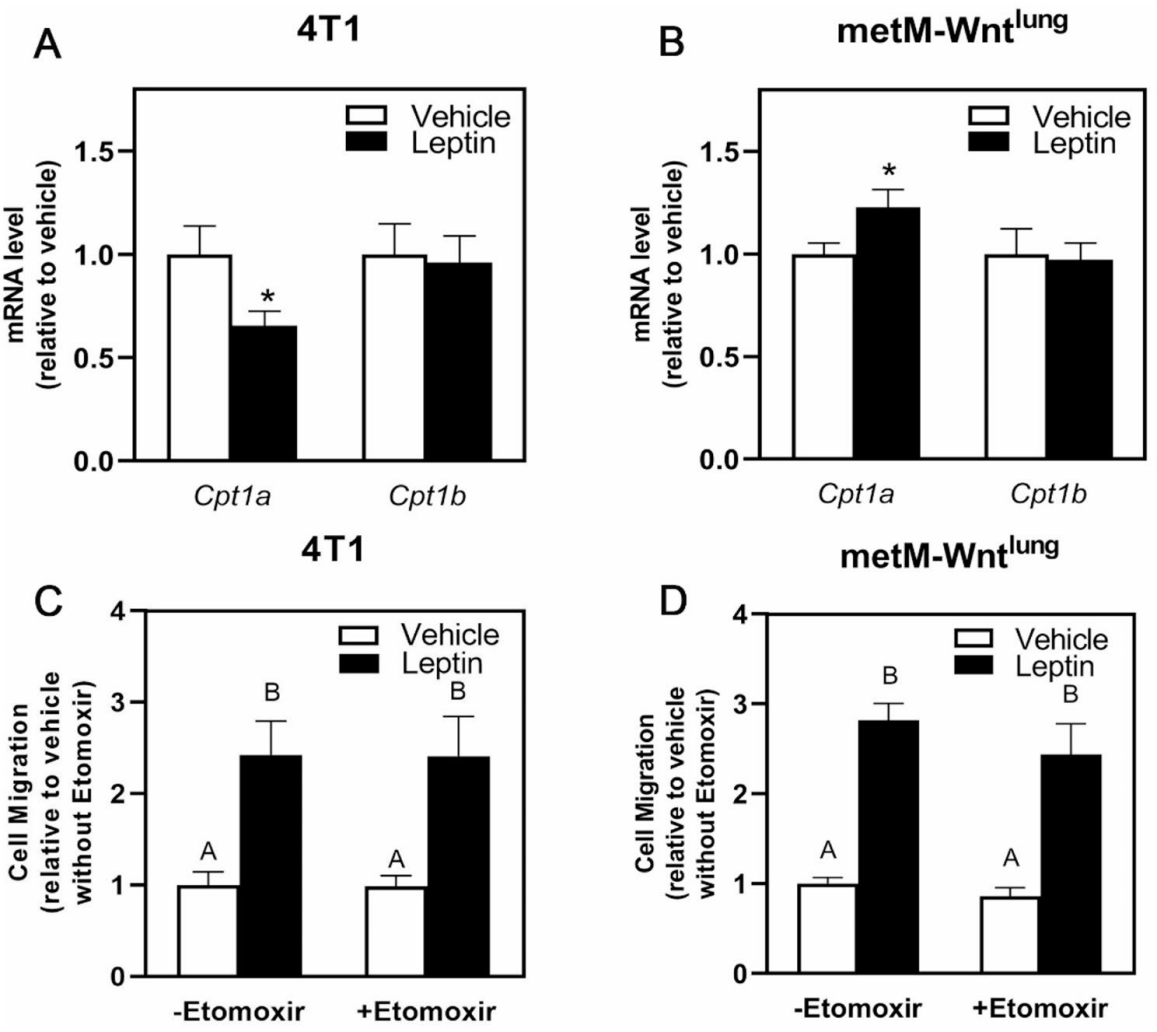
Role of Fatty Acid Oxidation in Leptin-Induced Migration in Metastatic Breast Cancer Cells. Following leptin treatment or vehicle, mRNA levels were measured in 4T1 (**A**) and metM-Wnt^lung^ (**B**) cells by qPCR. 4T1 (**C**) and metM-Wnt^lung^ (**D**) cells were pretreated with leptin before replating in serum free medium with or without etomoxir (75 µM) into transwell inserts. Values are mean ± SEM. Asterisk indicates a significant difference relative to vehicle (*p* < 0.05) of the same cell type. Groups with different letters are significantly different (*p* < 0.05) assessed by ANOVA

Because FAO has been shown to promote tumor cell migration [22], we investigated FAO’s contribution to the leptin-induced migration in these two cell lines. Relative to vehicle, leptin treatment decreased carnitine palmitoyltransferase 1a (*Cpt1a*), a rate-limiting enzyme FAO, mRNA level in 4T1 cells (Fig. 4A) but increased *Cpt1a* mRNA level in metM-Wnt^lung^ cells (Fig. 4B). Further, etomoxir, an inhibitor of CPT1, did not affect leptin-induced migration (Fig. 4C-D) in either 4T1 or metM-Wnt^lung^ cells, consistent with previous literature utilizing 25 mM glucose [13, 23]. These results suggest that FAO is not required for leptin-induced migration.

**Fig. 5:**
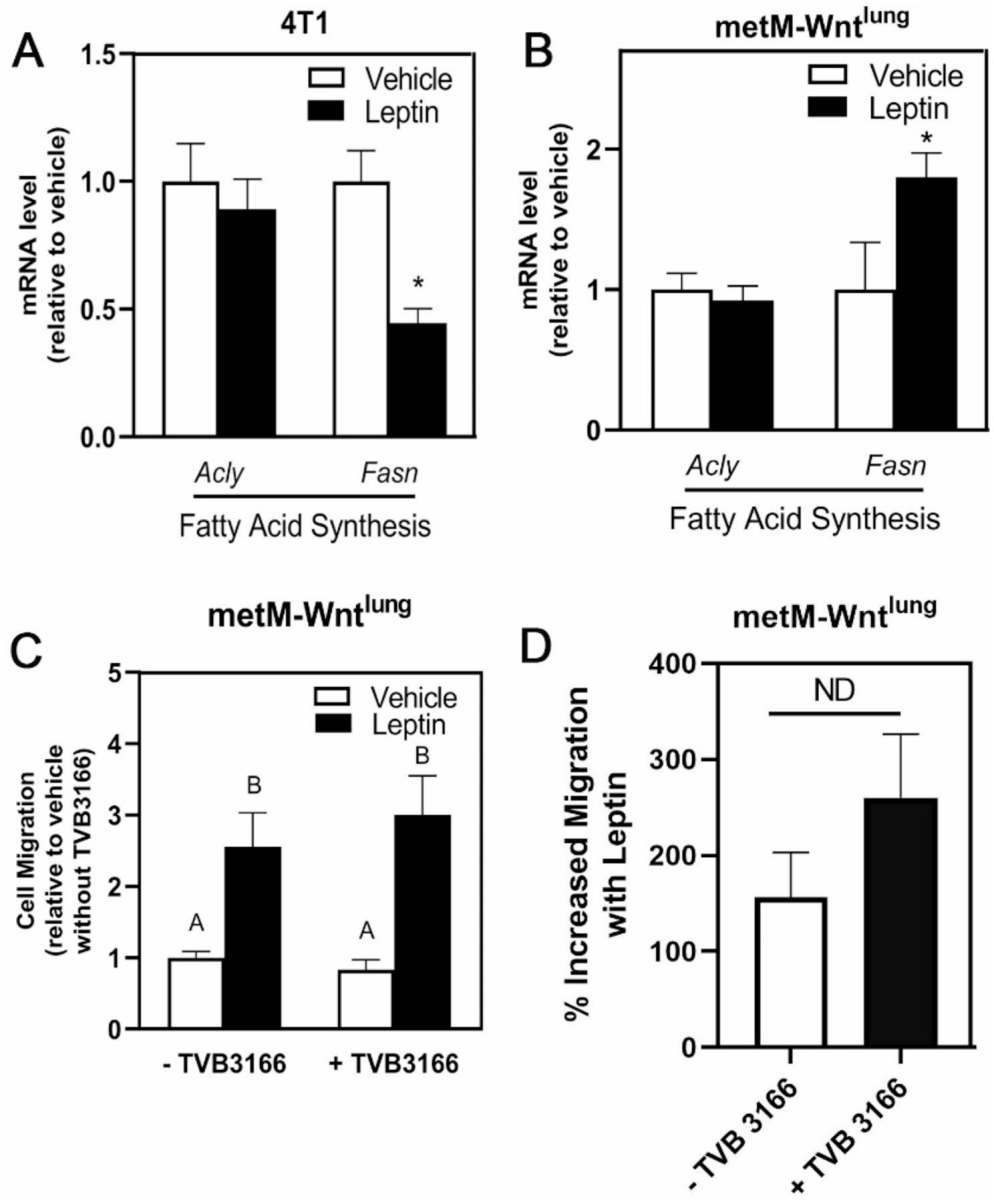
Effect of Inhibiting Fatty Acid Synthase on Leptin-Induced Migration in metM-Wnt^lung^ Cells. mRNA level of genes whose protein products are involved in fatty acid synthesis were measured in 4T1 (**A**) and metM-Wnt^lung^ (**B**) cells after treatment of leptin or vehicle by qPCR. metM-Wnt^lung^ cells were pretreated with leptin for 4 days before replating in serum free medium with or without the fatty acid synthase inhibitor TVB-3166 (42 nM) into transwell inserts (**C**). (**D**) In each group, the percent increased migration with leptin was calculated by the difference in migration per average of migration in vehicle. Values are mean ± SEM. Asterisk indicates a significant difference relative to vehicle (*p* < 0.05) of the same cell type. Groups with different letters are significantly different (*p* < 0.05) assessed by ANOVA. ND indicates no significant difference

To further explore the role of lipid metabolism in leptin-induced migration, we measured the expression of ATP citrate lyase (*Acly*) and *Fasn* following leptin treatment. *Fasn* mRNA level was suppressed by leptin treatment in 4T1 cells (Fig. 5A) but increased in the metM-Wnt^lung^ (Fig. 5B). However, inhibiting fatty acid synthase (TVB-3166) did not suppress leptin-induced migration in metM-Wnt^lung^ cells, indicating that fatty acid synthesis is not required for leptin-induced migration (Fig. 5C-D).

**Fig. 6:**
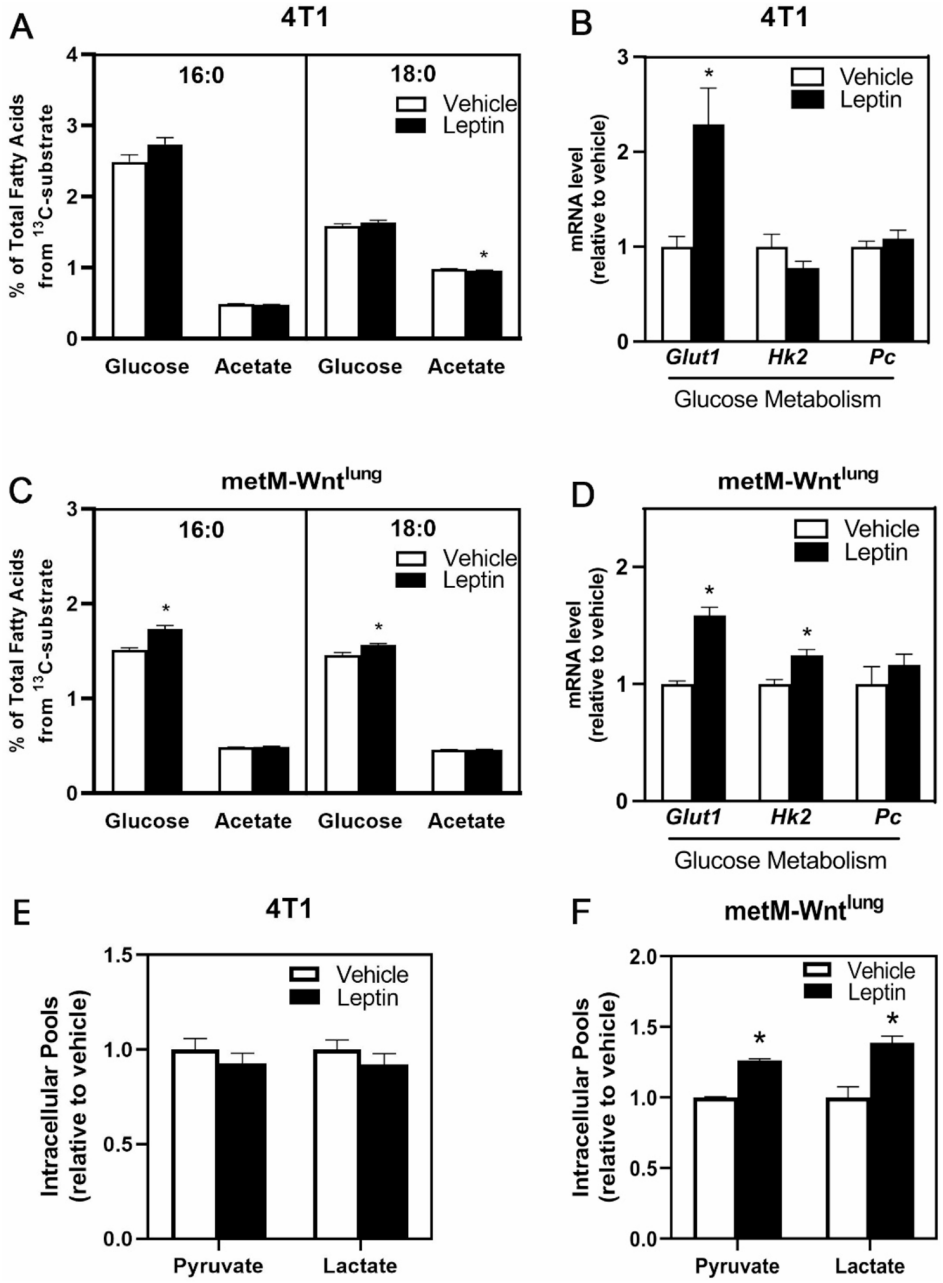
Leptin Effects on de novo Fatty Acid Synthesis in Metastatic Breast Cancer Cells. Conversion of glucose or acetate to palmitate or stearate in either vehicle or leptin-treated 4T1 (**A**) or metM-Wnt^lung^ (**C**) cells is represented as percent of total palmitate or stearate from each ^13^C substrate. Cells were treated with either vehicle or leptin for 4 days. In separate experiments, either [U]-^13^C-glucose or [U]-^13^C-acetate (24 h) incorporation into palmitate or stearate was analyzed using LC-MS/MS. Values are mean ± SEM. mRNA level of genes whose protein products are involved in glucose metabolism were measured in 4T1 (**B**) and metM-Wnt^lung^ (**D**) cells after treatment of leptin or vehicle by qPCR. Intracellular pools of pyruvate and lactate were measured by GC-MS/MS following leptin treatment in 4T1 (**E**) and metM-Wnt^lung^ (**F**) cells. Values are mean ± SEM. Asterisk indicates a significant difference relative to vehicle (*p* < 0.05)

Finally, we investigated the contribution of glucose metabolism to leptin-induced migration. Given that in some cancer cells glucose is the predominant source for fatty acids synthesis *de novo* [24], we measured carbon incorporation from [U]-^13^C–acetate and [U]-^13^C–glucose substrates into various fatty acids. First, basal fatty acid synthesis of palmitate from ^13^C–glucose was similar in 4T1 and the metM-Wnt^lung^ cells (4.61 ± 0.1 vs. 4.38 ± 0.15% of total (labeled and unlabeled) palmitate, respectively). Leptin treatment promoted palmitate and stearate synthesis from [U]-^13^C–glucose in metM-Wnt^lung^ but not 4T1 cells (Fig. 6A, C).

The mRNA level of glucose transporter 1 (*Glut1*), which facilitates glucose transport across the cell membrane, was increased with leptin treatment in both 4T1 and metM-Wnt^lung^ cells (Fig. 6B, D). Leptin treatment also increased hexokinase 2 (*Hk2)*, a key glycolytic enzyme, mRNA abundance, in metM-Wnt^lung^ cells (Fig. 6D). In addition, intracellular pool sizes of the end products of glycolysis, pyruvate and lactate, were measured after leptin treatment. In basal conditions, pyruvate pools size was 59% greater in metM-Wnt^lung^ cells compared to the 4T1 cells (*p* = 0.005, data not shown), with similar lactate pool sizes (*p* = 0.79) between the cell lines. Intracellular pool sizes of pyruvate and lactate were induced by leptin treatment in metM-Wnt^lung^ but not 4T1 cells (Fig. 6E-F), suggesting that glucose metabolism may be a critical regulator of migration in the metM-Wnt^lung^ but not the 4T1 cells.

**Fig. 7:**
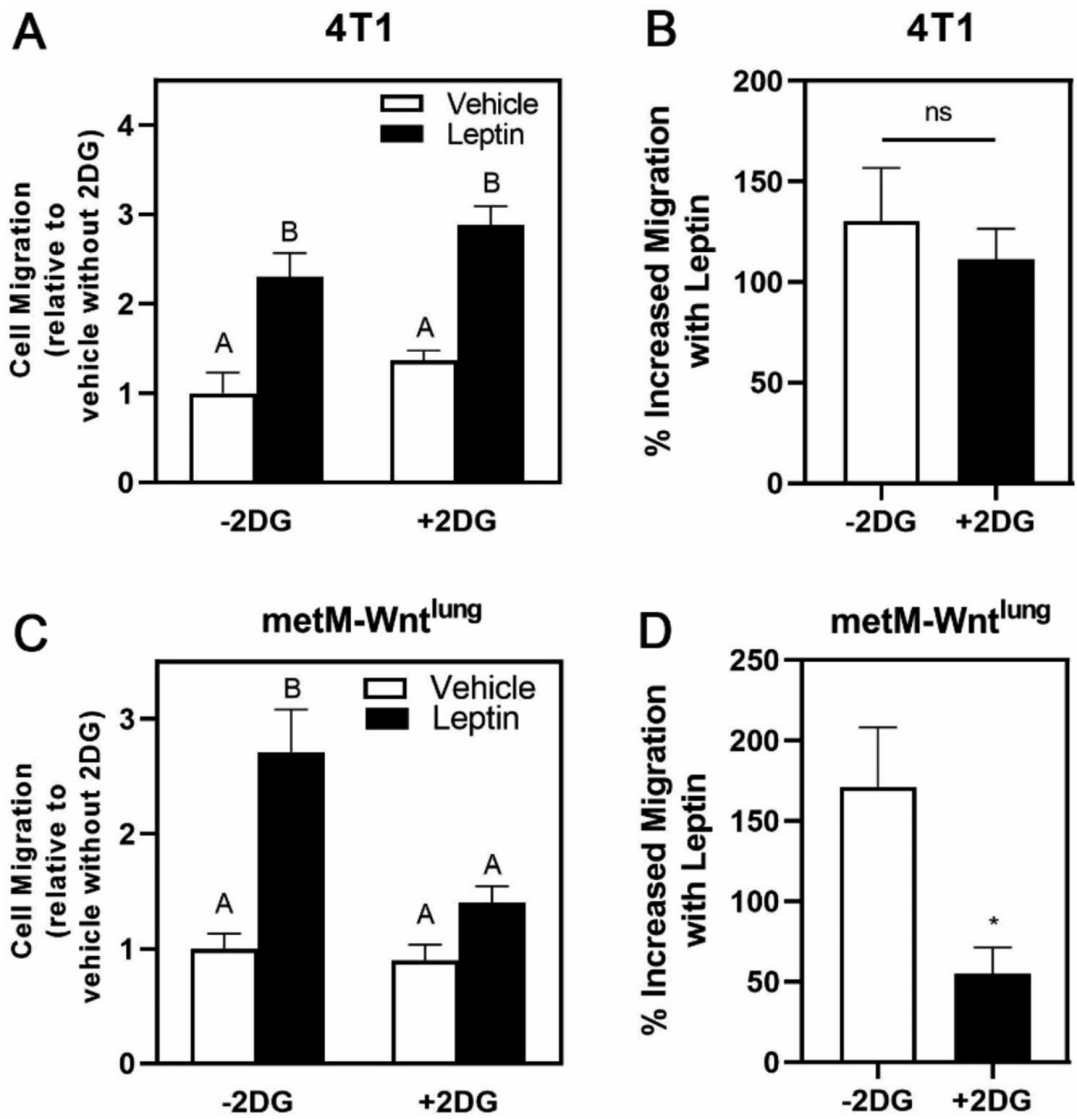
Role of Glycolysis in Leptin-Induced Migration in Metastatic Breast Cancer Cells. 4T1 (**A**, **B**) and metM-Wnt^lung^ (**C**, **D**) cells were pretreated with leptin before replating in serum free medium with or without 2-deoxyglucose (2DG) into transwell inserts and migration assessed. The dose of 2DG was 0.3 mM was employed for 4T1 and 5 mM of 2DG was used for metM-Wnt^lung^ cells. (**B**, **D**) In each group, the percent increase in migration with leptin treatment was calculated by the difference on migration per average of migration in vehicle. Values are mean ± SEM. Asterisk indicates a significant difference relative to vehicle (*p* < 0.05) of the same cell type. Groups with different letters are significantly different (*p* < 0.05) assessed by ANOVA. NS indicates no significant difference

Blocking glycolysis using 2DG (5 mM) potently suppressed leptin-induced migration in metM-Wnt^lung^ cells (Fig. 7C-D). Treatment of 4T1 cells with low concentrations (0.3 mM) of 2DG did not affect leptin-induced migration in 4T1 cells (Fig. 7A-B), but higher concentrations (2.5 and 5 mM) of 2DG induced cell death (data not shown), suggesting differential regulation of energy metabolism between the 4T1 and metM-Wnt^lung^ cells, with the latter requiring glycolysis for leptin-induced migration.

## Discussion

It has been reported that high glucose levels promote both the proliferation and migration of cancer cells [23]. In addition, Su et al., show that high glucose levels increase leptin signaling in fibrosarcoma cells through AMPK signaling [10], suggesting an important role of glucose on mediating leptin’s effects. However, the impact of physiological levels of glucose on leptin-mediated migration has not yet been elucidated. Therefore, we investigated the mechanism by which leptin increases migration in both 4T1 and metM-Wnt^lung^ cells when maintained in 5 mM glucose medium.

We show that *Ob-R* mRNA level was decreased with leptin treatment in 4T1 but was unchanged in metM-Wnt^lung^ cells. This is consistent with other research which demonstrated that while leptin increases invasive capability of MCF-7 and SKBR3 breast cancer cells, it does not influence protein levels of OB-R [25]. This suggests that different cancer cells respond differently to regulation of leptin receptor levels, and that these levels do not necessarily reflect the impact of leptin signaling regarding cancer-related outcomes. Changes in lipid metabolism are associated with cancer cell progression such as cell growth, apoptosis, and migration [26]. Fatty acids may provide substrates for energy via FAO as well as serve as signaling molecules. Fatty acids may be available from intracellular lipid stores, taken up into the cell, or synthesized endogenously. Several studies have demonstrated that leptin signaling alters lipid metabolism. For example, Palhinha et al., show that leptin induces neutral lipid storage levels assessed using BODIPY in 3T3-L1 preadipocytes [27]. In contrast, leptin increases lipolysis in rat adipose tissues [28]. However, the impact of leptin on fatty acid metabolism in breast cancer cells has not been investigated when cells are maintained in physiological concentrations of glucose. In our study, leptin treatment did not change TAG levels or fatty acid uptake in either 4T1 or metM-Wnt^lung^ cells (Fig. 3A-B). The latter result contrasts with previous work by Blanquer-Rosselló et al., which indicated that leptin increases FAT/CD36 protein levels in MCF-7 [15]. The discrepancy between our results may be due to the different cell types employed, their use of 25 mM glucose in the media compared to the 5 mM used in the current studies. It is also possible that 4T1 and metM-Wnt^lung^ cells do not require catabolism of fatty acids to provide energy following leptin treatment. The results of our study demonstrate that inhibiting FAO does not impact leptin-induced migration in 4T1 and metM-Wnt^lung^ cells, suggesting that FAO is not necessary for leptin-induced cell migration. This is consistent with other research that demonstrates that inhibiting FAO by etomoxir does not show any impact on invasion in E0771 breast cancer cells [13].

We also examined *de novo* fatty acid synthesis by utilizing [U]-^13^C–acetate and [U]-^13^C–glucose and measuring their incorporation into fatty acids to determine the pathway by which leptin may influence fatty acid synthesis. In 4T1 cells, leptin did not change carbon incorporation from glucose into either palmitate (16:0) or stearate (18:0). It is interesting to note, however, that leptin treatment increased mRNA levels of the glucose transporter *Glut1* while having no effect on at the mRNA level on the first enzyme of the glycolysis pathway, *Hk2. Fasn* mRNA level was decreased with leptin in 4T1 cells. These altered gene levels, increased *Glut1* and decreased *Fasn* following leptin treatment, may help explain the lack of difference in *de novo* fatty acid synthesis from glucose with leptin treatment. It is plausible that while glucose metabolism is increased, metabolic reprogramming may have occurred, where glucose is cycling through glycolysis without further metabolizing through the TCA cycle. Additionally, leptin significantly decreased acetate-derived carbon incorporation into stearate. Providing acetate as a substrate, which can bypass standard glucose metabolism pathways, supplies a direct substrate to produce acetyl-CoA for FA synthesis. This result is consistent with the observed decrease in *Fasn* mRNA level noted with leptin treatment. On the other hand, leptin increased glucose-derived carbon incorporation into both palmitate and stearate in the metM-Wnt^lung^ cells. In addition, mRNA levels of *Glut1*, *Hk2* and *Fasn* were increased in metM-Wnt^lung^ cells with leptin treatment, suggesting that these changes at the gene level may play a role in the leptin-induced increase on *de novo* fatty acid synthesis from glucose. Thus, although leptin does not affect fatty acid synthesis in 4T1 cells when maintained in 5 mM glucose, it does increase *de novo* fatty acid synthesis both upstream and downstream of the lipogenic pathway in metM-Wnt^lung^ cells, likely through the upregulation of key glucose metabolism enzymes and fatty acid synthase activity.

As *Glut1* mRNA levels were increased in both 4T1 and metM-Wnt^lung^ cells, we hypothesized that glycolysis is required for the leptin-induced migration observed. Inhibiting glycolysis by 2DG blunts leptin-induced migration in metM-Wnt^lung^ cells but does not have any effects on migration with leptin-induced 4T1 cell migration. The results in the metM-Wnt^lung^ cells differ from the findings of Blanquer-Rosselló et al., indicating leptin increases the utilization of fatty acids as fuel for energy instead of glycolysis in MCF-7 breast cancer cell lines [15]. This differential effect of leptin may be due to different cell types, similar to our differential results between the 4T1 and metM-Wnt^lung^ cell types. Thus, glucose metabolism requirement for leptin-induced migration varies in different metastatic breast cancer cells.

We also established that leptin increases AMPK activation by phosphorylation in 4T1 cells but inhibits AMPK activity in metM-Wnt^lung^, although our results demonstrate that these alterations do not play a role in leptin-induced migration in either cell line. The difference in AMPK activation is consistent with the varying mRNA levels of genes related to glucose metabolism, as well as *de novo* fatty acid synthesis from glucose following leptin treatment. In 4T1 cells, while *Glut1* mRNA levels were increased in response to leptin, *Fasn* mRNA levels were decreased. These gene-level changes may cumulatively eliminate effects on carbon incorporation from glucose into palmitate, as well as AMPK activation, in 4T1 cells. When AMPK is activated, downstream acetyl-CoA carboxylase (ACC) is inactivated by phosphorylation, leading to the reduction of malonyl-CoA generation for subsequent fatty acid synthesis. This is consistent with previous research that indicates direct inactivation of ACC by leptin leads to invasion of breast cancer cells [13]. On the other hand, in metM-Wnt^lung^ cells, mRNA level of *Fasn* and *Glut1* were both increased. In addition, palmitate synthesis from glucose was also increased with leptin treatment. Furthermore, intracellular pool sizes of the end products of glycolysis, pyruvate and lactate, were increased only in metM-Wnt^lung^ cells, not in 4T1 cells. This increase may lead to a sufficient energy status and inactivation of AMPK in metM-Wnt^lung^ cells. This differential regulation suggests that, despite the distinct genetic mutations, the AMPK network independently modulates cellular metabolism in response to leptin, highlighting a mechanism that is not solely dictated by genetic alterations. Together, these results suggest that leptin-mediated AMPK activation or inactivation is likely cell type-specific in response to differential glucose and lipid metabolism. Understanding these cell type-specific responses is crucial for unraveling the complexities of metabolic regulation and developing targeted therapeutic strategies.

## Conclusion

**Fig. 8:**
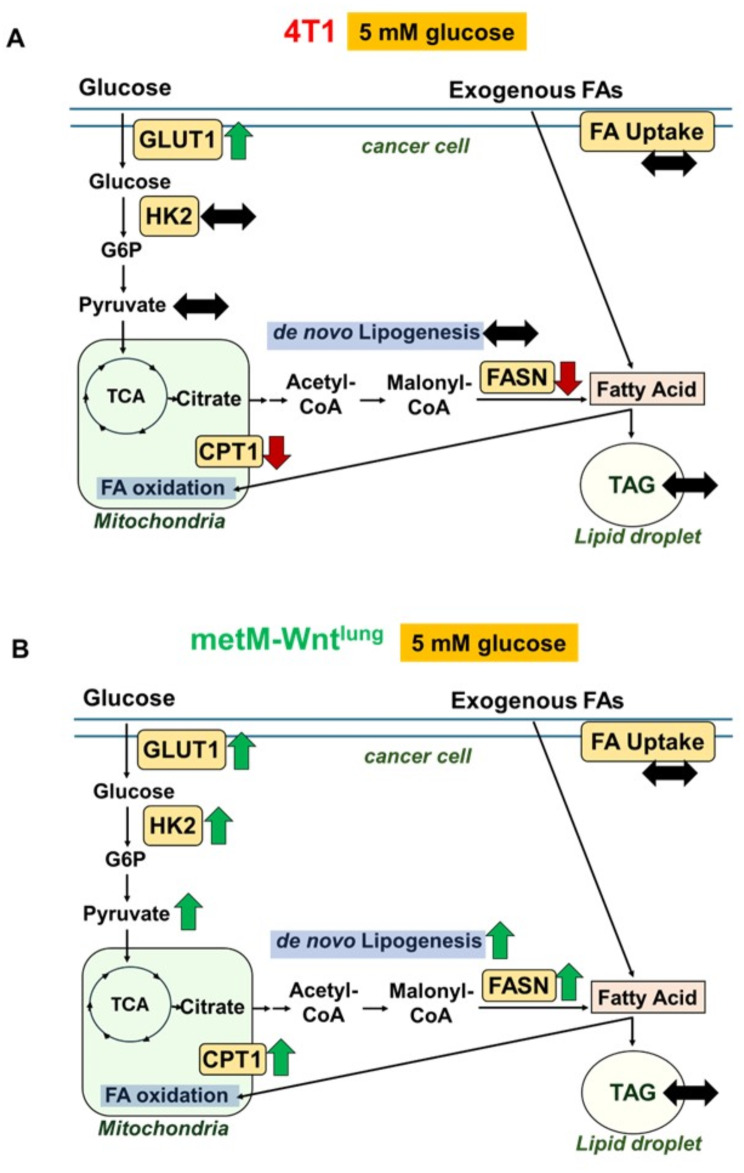
Summary of the Differential Effect of Leptin on Energy Metabolism in (A) 4T1 and (B) metM-Wnt^lung^ Metastatic Breast Cancer Cells. GLUT1: glucose transporter 1; HK2: hexokinase 2; G6P: glucose 6-phosphate; CPT1: carnitine palmitoyltransferase 1; FASN: fatty acid synthase; TAG: triacylglycerol; FA: fatty acid

Our study is the first to investigate the impact of leptin on metastatic breast cancer cell migration maintained in a physiological level of glucose. The results of the current study demonstrate increased migratory capability of breast cancer cells following leptin treatment in physiological glucose conditions, an interesting result not reported previously (Fig. 8). We also demonstrate that leptin affects energy metabolism differentially, activating AMPK in 4T1 and inactivating AMPK in metM-Wnt^lung^ cells, although AMPK activity does not mediate the leptin-induced migration in either cell line. Overall, FAO is not involved in leptin-induced migration, although fatty acid synthesis from glucose is increased only in the metM-Wnt^lung^ cells. Additionally, we illustrate that glycolysis is involved in leptin-induced migration in metM-Wnt^lung^, but not in 4T1 cells. Further studies are necessary to elucidate the mechanism in each cell type underlying this effect of leptin to pinpoint how these mechanisms vary across different cell types, which may provide insight to help uncover novel therapeutic targets and strategies to combat disease states such as obesity and cancer.

## Data Availability

No datasets were generated or analysed during the current study.

## Abbreviations

TNBC: Triple Negative Breast Cancer
Ob-R: Leptin Receptor
AMPK: AMP-activated protein Kinase
FASN: Fatty Acid Synthase
CPT1a: Carnitine Palmitoyltransferase 1a
GLUT1: Glucose Transporter 1
JAK: Janus Kinase
STAT: Signal Transducer and Activator of Transcription
MAPK: Mitogen-Activated Protein Kinase
FA: Fatty Acids
FAO: Fatty Acid Oxidation
DMEM: Dulbecco’s Modification of Eagle’s Medium
FBS: Fetal Bovine Serum
AICAR: 5-Aminoimidazole-4-Carboxamide Ribonucleotide
2DG: 2-Deoxyglucose
BSA: Bovine Serum Albumin
TAG: Triacylglycerol
BCA: Bicinchoninic Acid
AUC: area under the curve
RIPA: Radioimmunoprecipitation Assay
ACLY: ATP Citrate Lyase
HK2: Hexokinase 2
ACC: Acetyl-CoA Carboxylase
